# Complications in Idiopathic Pulmonary Fibrosis: Focus on Their Clinical and Radiological Features

**DOI:** 10.3390/diagnostics10070450

**Published:** 2020-07-03

**Authors:** Federica Galioto, Stefano Palmucci, Giovanna M. Astuti, Ada Vancheri, Giulio Distefano, Francesco Tiralongo, Alessandro Libra, Giacomo Cusumano, Antonio Basile, Carlo Vancheri

**Affiliations:** 1 Radiology Unit 1, Department of Medical Surgical Sciences and Advanced Technologies—University Hospital “Policlinico-Vittorio Emanuele”, University of Catania, Via Santa Sofia n. 78, 95123 Catania, Italy; federicagalioto91@gmail.com (F.G.); giovannam.astuti@gmail.com (G.M.A.); giuliodistefano@gmail.com (G.D.); tiralongofrancesco91@hotmail.it (F.T.); basile.antonello73@gmail.com (A.B.); 2 Regional Centre for Interstitial and Rare Lung Disease, Department of Clinical and Molecular Biomedicine, University of Catania, 95123 Catania, Italy; adact1@hotmail.it (A.V.); alessandrolibra@outlook.it (A.L.); giacomare55@hotmail.com (G.C.); vancheri@unict.it (C.V.)

**Keywords:** interstitial lung diseases, HRCT, IPF, ILD, lung cancer

## Abstract

Idiopathic pulmonary fibrosis (IPF) is a fibrotic lung disease with uncertain origins and pathogenesis; it represents the most common interstitial lung disease (ILD), associated with a pathological pattern of usual interstitial pneumonitis (UIP). This disease has a poor prognosis, having the most lethal prognosis among ILDs. In fact, the progressive fibrosis related to IPF could lead to the development of complications, such as acute exacerbation, lung cancer, infections, pneumothorax and pulmonary hypertension. Pneumologists, radiologists and pathologists play a key role in the identification of IPF disease, and in the characterization of its complications—which unfortunately increase disease mortality and reduce overall survival. The early identification of these complications is very important, and requires an integrated approach among specialists, in order to plane the correct treatment. In some cases, the degree of severity of patients having IPF complications may require a personalized approach, based on palliative care services. Therefore, in this paper, we have focused on clinical and radiological features of the complications that occurred in our IPF patients, providing a comprehensive and accurate pictorial essay for clinicians, radiologists and surgeons involved in their management.

## 1. Introduction

Idiopathic pulmonary fibrosis (IPF) is a debilitating and fatal scarring lung disease, with poor prognosis. It represents the most common form of interstitial lung disease (ILD), and it has been defined as a chronic, progressive fibrosing interstitial pneumonia of unknown cause with a usual interstitial pneumonia (UIP) pattern depicted on HRCT imaging or at histology [1]. It is considered a rare disease—occurring in less than 5 per 10,000 person-years. In Europe, approximately 40,000 new cases are diagnosed each year.

Clinically, IPF disease is characterized by respiratory alterations, such as dyspnea or decreased pulmonary function; pulmonary function tests reveal a restrictive pattern. Clinical symptoms, at the beginning, may be misinterpreted, so that patients are wrongly considered as affected by chronic bronchitis [2]. IPF diagnosis needs a multidisciplinary evaluation, which should be based on a correct collection of clinical history, with particular regard to exposure, tobacco smoking and signs of connective tissue disease [3,4]. Radiologists and pathologists play a key role in identifying IPF disease and its complications. In fact, the progression of fibrosis may lead to the development of several complications, mainly represented by lung cancer, acute exacerbation, pulmonary hypertension, pneumothorax and infections. Due to these complications, IPF mortality increases, and the overall survival remains—despite the introduction of antifibrotic treatment—at about 3–5 years [5].

The early identification of these complications is crucial, in order to increase the survival of the disease; in addition, the results of recent genetic studies, in combination with clinical and radiological features, are trying to introduce some biomarkers for monitoring disease [6].

Therefore, the aim of this paper is to illustrate clinical and radiological features of IPF complications, in order to provide useful skills and suggestions, and to achieve their early diagnosis and management.

## 2. Methods

### 2.1. Study Design, Setting and Participants

Through the PubMed database, an extensive search was performed in the fields of IPF and its complications. In our research, we have included only articles in English for which it was possible to access the entire content; we excluded recurrent articles from the same authors and articles written in other languages.

We have analyzed complications which occurred in our IPF population, selecting cases from January 2013 to December 2019; more in detail, patients have been selected from our radiology information system (RIS) archive and from the database of the Regional Centre for Interstitial and Rare Lung Disease of our hospital. For each IPF patient, we have retrospectively evaluated clinical history, imaging data and morphological data.

Since not all patients performed radiological examinations in our Radiology Institute, we have considered clinical cases having chest CT scans that were compatible with HRCT standard protocol [7], according to the following technical parameters: volumetric scan acquired at full inspiration; thin-section CT images ranged between 0.625 and 1.5 mm; sharp kernel imaging reconstruction; contiguous or overlap images; and no contrast media administration.

After a qualitative evaluation of CT examinations, all patients were subdivided into the following different subgroups based on their different complications: 1. lung cancer; 2. acute exacerbation; 3. pulmonary hypertension; 4. pneumothorax; 5. infections; and 6. other.

### 2.2. Statistical and Data Analysis

Data were collected on a Microsoft Excel database (Microsoft Corporate, Redmond, WA, USA). Continuous variables were presented as means (standard deviations), and categorical variables were presented as percentages. The authors had full access to the data and take full responsibility for their integrity.

## 3. Results

In our study, exploring our database from January 2013 up to December 2019, 450 patients have received the diagnosis of IPF according to the diagnostic criteria ATS and ESR 2000 [8]: in this population, we have focused on patients who developed complications.

Considering lung cancer (LC), from this IPF database, we have subselected only subjects having at least two HRCT scans in our database; therefore, a total of number of 212 patients with IPF was finally investigated. Among this subgroup, LC was found in 19 patients (M: 14, 69.7 ± 8.3). At one year after the diagnosis of this complication, some of these patients died, in a number of 7 patients over 19 (showing a mortality rate of 36.85%).

**Figure 1:**
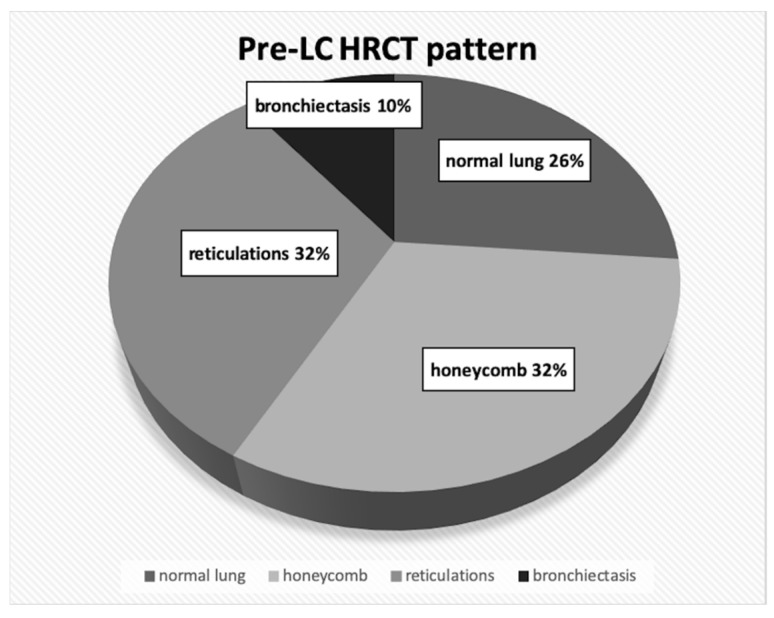
Prevalence of different HRCT patterns identified in our idiopathic pulmonary fibrosis (IPF) patients having lung cancer.

For each patient with LC, we retrospectively observed the HRCT examination acquired before tumor development: in 14 patients (73.7%), the tumor was found in pulmonary parenchyma having a fibrotic background, whereas in the other five patients, it was depicted as remote from fibrotic areas (26.3%). More in detail, in the 14 cases having LC in a fibrotic background, the morphological pattern was analyzed: in 6 out of 14 patients (32%), the tumor grew in honeycombing areas, in 6 patients (32%) in the lung parenchyma with reticulations and in the remaining 2 cases (11%), in pulmonary areas, with bronchiectasis (Figure 1).

**Figure 2:**
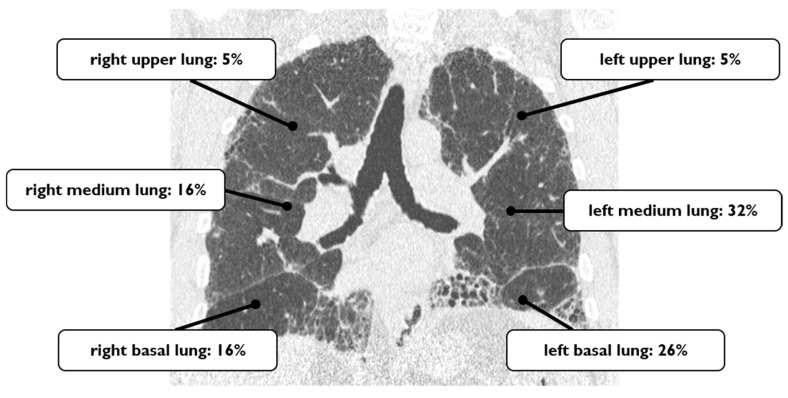
The coronal HRCT image shows the lung cancer distribution identified in our series.

The LC location found in our 19 cases has been schematically drawn, as reported in Figure 2. We have divided each lung into three regions (upper region—from apex to carena, medium region—from carena to inferior pulmonary veins and basal region—from inferior pulmonary veins to diaphragm). Considering this schematic repartition of the pulmonary zones, we found three tumors in the right basal lung, three tumors in the medium zone and only one tumor in the superior right pulmonary area (16%, 16% and 5%, respectively). In the left hemithorax, LCs distributed in the following way: five cases in the basal regions, and six cases in the medium portions of the lung; only in one patient was LC found in the superior zone (26%, 32% and 5%, respectively). Overall, we observed 12 cases in the left lung and 7 cases in the right lung (Figure 2).

Related to other common complications of IPF disease, in our database (*n* = 450 patients, from January 2013 up to December 2019), we found 23 patients who developed acute exacerbation (M: 20, 73.5 ± 9.2) with a mortality rate equal to 73%.

In this subgroup, 16 patients (69%) were smokers with a risk ratio of acute exacerbation development related to cigarette smoking of 2.1. Considering the presence of other comorbidities, 8 patients (35%) over 23 have GERD treated with proton-pump inhibitors (PPIs); 5 patients (22%) had professional exposure to dust and environmental pollutants; 1 patient was affected by allergies to aeroallergens; 1 patient reported exposure to birds for over three years before diagnosis, and finally, 14 patients (60%) had hypertension, with a risk ratio of 2 to acute exacerbation development.

Furthermore, in our database, we found 11 patients with a certain IPF diagnosis and pulmonary hypertension (PH) (M: 9, 70.1 ± 8.3). Among these patients, we observed an enlargement of the right pulmonary artery diameter, with a medium range of 30.2 mm ± 4.8 (normal value = 20 mm), and a mild enlargement of the left pulmonary artery diameters, with a medium range value of 27 mm ± 1.4 (normal value <20 mm). We also observed a mild enlargement of the pulmonary trunk, with a medium range of 34 mm ± 3.1 (normal value = 30 mm).

Only two male patients, aged between 68 and 70 years, reported an episode of pneumothorax (PNX) as a complication.

About pulmonary infections related to IPF, in our database, we found only one patient with a mycotic infection (man, 80 years old, ex-smoker, 1-pack year).

## 4. Discussion

IPF has a variable clinical course: many patients may remain stable over time, while others may experience relatively rapid deterioration and develop some complications [9].

These conditions may complicate the clinical course of the disease—increasing morbidity and mortality; for this reason, an early recognition and an accurate identification should be mandatory to provide the possibility of treating causes of worsening symptoms—as soon as they are diagnosed [10]—and sometimes to manage the patient in a personalized approach.

Hereby, we focused on the main clinical conditions that may be responsible for the acute deterioration of IPF, or that may significantly influence disease progression; as previously reported, these conditions may be summarized as the following:

- Lung cancer;
- Acute exacerbation;
- Pulmonary hypertension;
- Pneumothorax;
- Pulmonary infection.

### 4.1. Lung Cancer

IPF patients have an almost five times greater risk of LC compared with the rate observed in the general population [11]; the high risk of cancer is related to progressive and irreversible lung fibrosis caused by IPF disease. Studies have suggested that IPF and LC exhibit similarities and pathogenetic mechanisms in common, mainly represented by epigenetic and genetic abnormalities and uncontrolled proliferation mechanisms, irregular signaling pathways and abnormal expression of microRNAs [12,13,14].

Several factors may increase the risk of LC in patients with IPF: age, gender and smoking status are strongly linked to the development of cancer [15,16,17].

Pathogenesis can be based on inflammatory mediators with repeated episodes of cell damage and lesions to the respiratory epithelium, developing cellular atypia, metaplasia, dysplasia and invasive carcinoma [18]. In some studies, as reported in the paper published by Aubry et al. and in the paper written by Kishy et al., most cancers arise in a peripheral basal position having more severe fibrosis, or they develop at the junction of the normal lung with fibrosis [16,19,20,21]. In the review published by Ballester in 2019, LC was associated with honeycomb lesions, and they frequently develop from honeycomb areas or in the border between honeycombing and non-fibrotic areas, and epithelial metaplasia [22]. In addition, the authors state that in IPF patients having LC, more frequently, squamous metaplasia, but not cuboidal cell metaplasia or bronchial cell metaplasia, have been observed. Based on these findings, it was speculated “that it might reflect a constitutional susceptibility of IPF patients of developing lung carcinoma”—as reported in the same review written by Ballester [22].

Therefore, this tumor location is strongly related to the distribution of fibrosis, which could be considered to have a key role in the development of cancer.

In other studies, i.e., the study conducted by Park et al. in 2001, LC was equally distributed in the upper and lower lobes (52% and 48%, respectively), and the tumor was predominantly found in non-fibrotic areas (56%) [17,19].

Considering the histotype of lung tumor, Hironaka et al. [23] reported squamous cell carcinoma as the most common type of cancer in IPF, and this result accords to other similar reports [16,17,23,24,25]; others authors have reported that adenocarcinoma is the most common histological tumor [23,26]. In a recent paper written by Tomassetti et al., in a series of 181 patients with IPF, peripheral squamous cells (9.39%) and adenocarcinomas (8.35%) were the two most common histological types of LC [27]. In the study published by Ozawa et al. [28], in a population of 21 IPF-LC patients, squamous cell carcinoma was found in 38% of cases, and adenocarcinoma was reported with a slightly inferior percentage of cases, about 29%.

LC symptoms include hemoptysis, unintentional weight loss and constitutional symptoms, which may be minimal or non-specific

**Figure 3:**
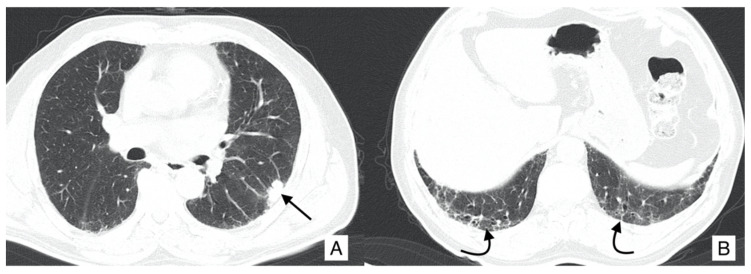
Axial HRCT images of a patient with IPF and lung cancer (LC). In (**A**), a lobulated lesion is depicted (black arrow), located at the periphery of the left lung. In (**B**), reticulations and bronchiectasis are clearly shown (black curved arrows), resembling a usual interstitial pneumonitis (UIP) probable pattern.

On HRCT, LCs that occur in IPF patients may have different appearances: it could be depicted as a well-defined rounded lesion, or it can develop as an air-space consolidation area; in other cases, it may be found as a nodular or lobulated lesion (Figure 3), and differential diagnosis from other entities is not easy. Usually, LC develops in the fibrotic area, in basal regions and it is associated with the presence of peripheral honeycombing [24,29]. Invasive adenocarcinoma and invasive mucinous adenocarcinoma may be detected as a ground-glass area having nodular consolidation inside [30].

**Figure 4:**
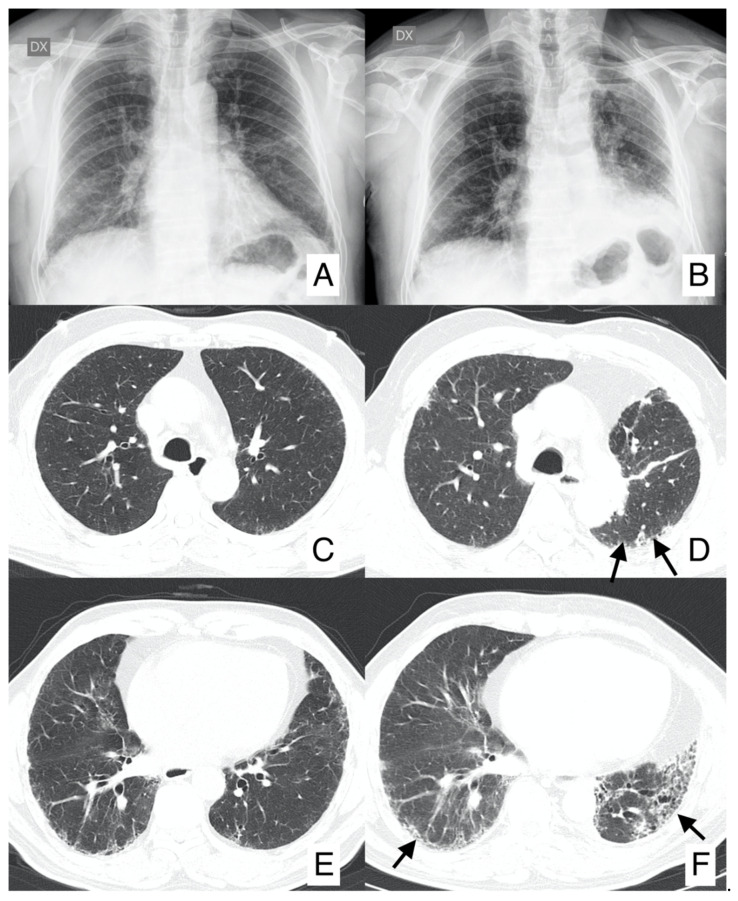
Progression of IPF disease after surgical resection of nodular neoplastic lesion (same patient as Figure 3, having IPF complicated by squamous cell carcinoma). Chest radiographs (**A**,**B**) acquired before and after surgical resection: it is possible to appreciate the increased reticular pattern (namely in the left lung). Axial CT images before (**C**,**E**) and after surgery (**D**,**F**). CT images clearly show progression of reticulations—well-depicted in subpleural regions (black arrows).

Surgical resection of early-stage cancers may be curative, even if due to IPF severity and its clinical evolution it may increase the risk of postoperative morbidity and mortality; surgical treatment has been considered a trigger of the development of acute exacerbation or may lead to disease progression (Figure 4). Surgical prognosis is improved by performing sublobar lung resections, reducing fluid overload, avoiding pulmonary hyperinflation, using high-flow oxygen therapy during surgery and using prophylactic antibiotic treatment [31]. Even radiotherapy or chemotherapy may increase the risk of exacerbations, especially by using docetaxel [32]. Studies have shown that the association of pirfenidone and nintedanib with chemotherapeutic agents may improve the clinical outcome, even if future researches are needed [31,32,33,34].

In the management of IPF patients, a critical issue is still represented by the recognition of a nodule in the pulmonary parenchyma: this event may happen during the follow-up of a stable disease, as occurred in the clinical example we have discussed. Diagnosis and management of these patients, who present a nodule on CT scans acquired during follow-up of IPF disease, have been recently evaluated and discussed by an expert panel [35].

According to the paper published in the literature by Tzouvelekis et al., the diagnosis and the management have been defined in the evaluation of the pulmonary nodule in IPF patients. The ATS/ERS guidelines published in 2013 recommend yearly HRCT in IPF patients, and the main reason to perform the follow-up examination is represented by the risk of LC.

In case of a pulmonary nodule found at follow-up HRCT, the long and short diameters need to be measured in order to obtain the medium diameter of the nodule found; according to the suggestions provided by the panel expert, three scenarios could be encountered by a radiologist:

- In case of an IPF patient having a nodule of <8 mm, an HRCT is needed every three–six months with continuous surveillance. If the HRCT demonstrates progression of the nodule, a PET-CT is recommended;
- In case of an IPF patient having a nodule of at least 8 mm, a PET-CT scan is highly recommended. If the PET reports a great uptake, the panel expert state that “minimally invasive procedures are needed, such as TTNB (Transthoracic needle biopsy) or a CT-TTNB for peripheral lesions or endobronchial ultrasound-guided transbronchial needle biopsy (EBUS-guided transbronchial needle biopsy) if there are pathological lymph nodes (≥8 mm)” [35]. If biopsy could be unsafe for the patient, or not indicated for the clinical context, “it is suggested a multidisciplinary and personalized approach”;
- In case of a patient having an advanced lesion on the HRCT (mass or nodule greater than 8 mm), a multidisciplinary and personalized approach is needed; based on the clinical conditions of the patient, in these cases, the panel experts state that “no further diagnostic procedures could be adopted” [35]—planning a personalized approach, based also on palliative support.

**Figure 5:**
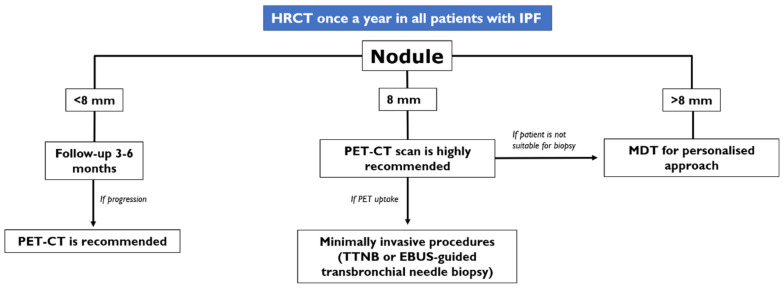
Management of a new nodule detected on follow-up HRCT, according to the approach suggested in the article published by Tzouvelekis et al. [35].

These criteria for the management of a new nodule detected in follow-up HRCT are summarized in Figure 5 [35].

**Figure 6:**
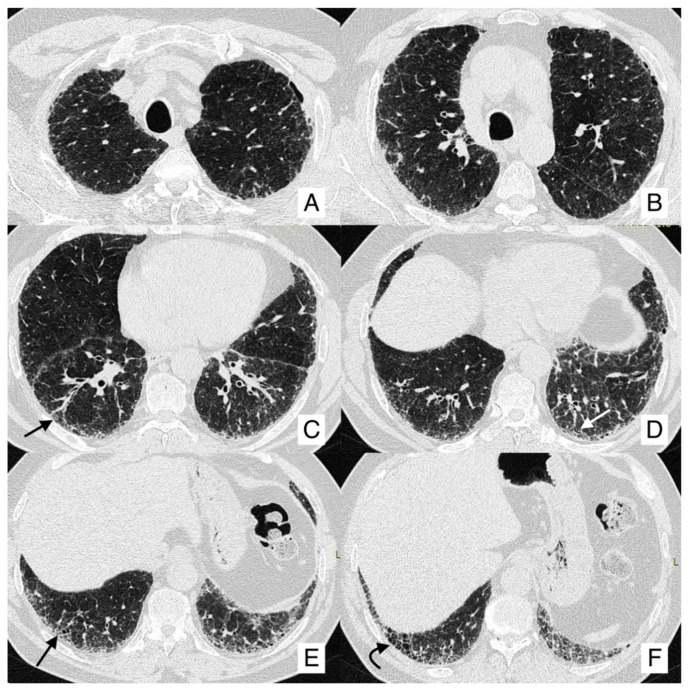
A patient with typical HRCT features of IPF. Images acquired at three different levels of the thorax (**A**,**B**) obtained above the carena; (**C**,**D**) scans obtained at middle levels of lungs; (**E**,**F**) acquired at basal regions. Reticulations are located in peripheral regions, more represented in the medium regions of the lung, and at the pulmonary basis (black arrows); there is a typical cranio-caudal gradient of these imaging findings. Cystic spaces are well-demonstrated (honeycomb area is clearly recognizable in the right costophrenic angle, black curved arrow in (**E**), reticulations and small bronchiectasis are well-appreciated (black arrows in (**C**,**E**), white arrow in (**D**). Therefore, a UIP pattern could be recognized.

**Figure 7:**
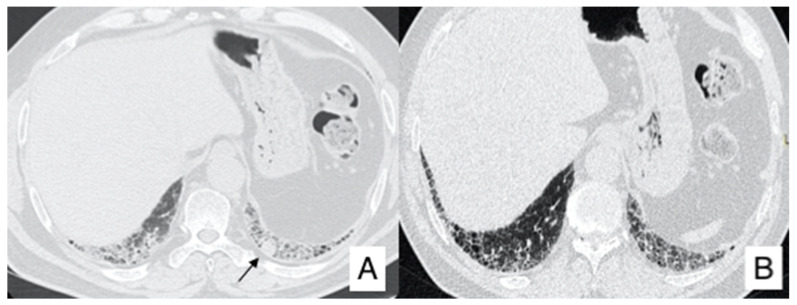
One year later, follow-up HRCT of the same patient in Figure 6, identified a nodule in the left lower lobe (black arrow in (**A**), that was not shown in the first examination (**B**).

**Figure 8:**
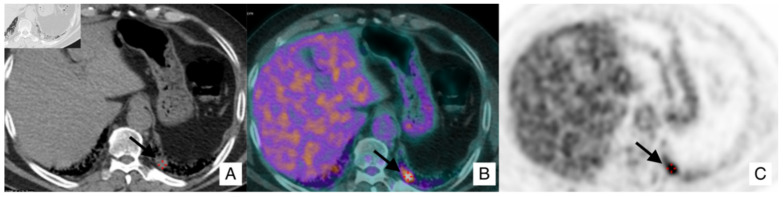
PET-CT (**A**–**C**) of the same patient of Figure 6 and Figure 7. The exam demonstrates an increased activity (uptake of 6.84) in the nodular lesion, which is clearly depicted (black arrow) in the axial CT image (**A**), in the fusion colour image (**B**) and in the black and white PET map (**C**).

Figure 6, Figure 7 and Figure 8 summarize the clinical experience of a patient with IPF and LC.

This 66-year-old IPF male patient followed at our Regional Center for Interstitial Lung Disease was investigated by HRCT, showing a typical UIP pattern, with honeycombing, subpleural reticulations and traction bronchiectasis (Figure 6).

One year later, follow-up HRCT identified a nodule in the left lower lobe, that was not shown in the first examination (Figure 7). For this reason, a PET was required, demonstrating the presence of an area of increased activity and uptake (Figure 8).

We discussed this case in our multidisciplinary meeting, and a thoracic surgeon was involved in the discussion. The first decision was to characterize the nodule, performing a fine needle agobiopsy (FNAB); unfortunately, the procedure was not able to provide adequate material for a histological diagnosis. Therefore, the next step proposed was surgical intervention; an atypical resection was performed, and a final diagnosis of “squamous carcinoma” was achieved.

### 4.2. Acute Exacerbation

According to the definition reported in the literature by the “International Working Group Report”, acute exacerbation of IPF is “an acute worsening of respiratory symptoms with new opacities on HRCT images without other identifiable causes” [36]. This exacerbation may occur at any time during the course of IPF disease, and for some patients, it may be also the first manifestation of the disease [37].

It is not clearly linked to age or smoking history, but it seems to be more frequent in men; surgical lung biopsy has been demonstrated to be a risk factor, even if it is difficult to distinguish these cases from post-surgical acute respiratory distress syndrome (ARDS) [38,39,40].

Acute exacerbations of IPF are commonly encountered in patients with low Forced Vital Capacity (FVC), Diffusion lung CO (DLCO), low six-minute-walk distance, pulmonary hypertension, poor baseline oxygenation, increased dyspnea and other cardiovascular disease or high body mass index [41], and furthermore, an elevated serum level of Krebs von Lungen-6 (KL-6) [42].

The etiology of acute exacerbation of IPF is unknown, but there are several hypotheses. First of all, it may represent a pathobiological manifestation of the disease process characterized by idiopathic lung injury. Second, it may be caused by several biologically distinct conditions undiagnosed, such as viral infections (e.g., herpesviruses) [43,44,45,46,47], aspiration of gastric contents (with diffuse alveolar damage) and acute worsening because of gastroesophageal reflux [48,49,50,51]. Third, exacerbation may be considered the sequelae of an acute stress to the lung, leading to an acceleration of the already abnormal fibroproliferative process intrinsic to IPF [52].

The pathobiology of an episode of acute exacerbation of IPF may be linked to intrinsic biological dysfunctions of the lung with IPF disease, such as those related to disordered epithelial cell integrity, cellular inflammation, cytokines, matrix metalloproteinases (MMPs) and coagulation components, whose patients are more susceptible to external insults [36]. Even genetic predispositions, mainly represented by mutations related to surfactant proteins genes, or which regard to the telomerase reverse transcriptase (hTERT) and/or RNA component (hTR) genes encoding telomerase components, have been involved in the development of frequent exacerbations. Clinical manifestations include the onset of new dyspnea and symptoms such as cough, fever, flu-like [53,54,55], severe hypoxemia and respiratory failure, which requires mechanical ventilation, are commonly found in these patients. Imaging is used to evaluate acute exacerbation in patients with IPF; chest X-rays may reveal a reticular shadow on the lower lobes, obviously better appreciated in the comparison with previous films [56]. HRCT has been introduced in the IPF guidelines, due to the fact that it may reveal the coexistence of the IPF model with the appearance of new parenchymal opacities—after an accurate exclusion of alternative causes (for example, infections, left heart failure or identifiable lung lesions) [57]. According to the paper published by Akira et al. in 2008, three patterns of acute exacerbation have been described: peripheral, multifocal and diffuse parenchymal opacifications (ground-glass opacities or consolidations) [58]. All these CT findings may occur on the background of pre-existing IPF patterns. Multifocal opacifications with patchy involvement of both central and peripheral regions may progress to a diffuse degree of opacification, identifying the multifocal pattern as an earlier stage of acute exacerbation than the diffuse form [58]; instead, the peripheral pattern may not progress to diffuse opacification. The three CT patterns are strongly connected to the outcome of patients, having a prognostic value: the diffuse pattern is linked to a higher risk of death than the multifocal or peripheral patterns [10,58].

**Figure 9:**
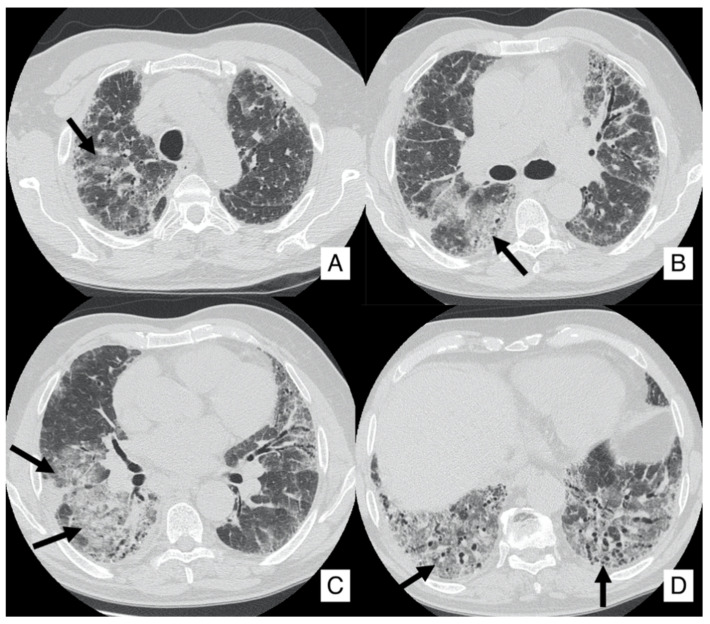
Acute exacerbation of IPF in an 80-year-old man (**A**–**C**). These CT scans demonstrate an increased diffuse ground-glass opacity with an increased parenchymal density, especially in the right upper lobe (black arrows in **A** and **B**) and in the upper part of the right inferior lobe (black arrows in **C**). At the pulmonary bases, traction bronchiectasis is clearly recognized (black arrows in **D**). This increased GGO pattern is related to an acute exacerbation of IPF. At clinical evaluation, a decreased vesicular murmur and wheezes in the basal-middle areas were found.

From our case series, we extract and comment on the clinical history and imaging of an IPF patient, who had experienced an episode of acute exacerbation. More in detail, we report clinical and radiological features of a non-smoker with 79 years, with no birds exposure, but with allergies to aeroallergens and allergic asthma, diagnosticated 20 years before. He has hypertension pharmacologically treated. In 2018, he arrived at our hospital with a suspected pulmonary interstitial disease. Previous chest CT scans clearly demonstrated traction bronchiectasis and small honeycomb areas in bilateral lower lobes, subpleural reticulations and diffuse peri-bronchovascular thickness; at the pneumological evaluation, clinical features as dyspnea and alterations were found. About one year later, he complained of clinical worsening, and he underwent a new chest CT in March 2019. This CT demonstrates an increased diffuse ground-glass opacity with an increased parenchymal density, especially in the right upper lobe and in the upper part of the right inferior lobe (Figure 9). This increased GGO pattern is related to an acute exacerbation of IPF. At clinical evaluation, a decreased vesicular murmur and wheezes in the basal-middle areas were found.

**Figure 10:**
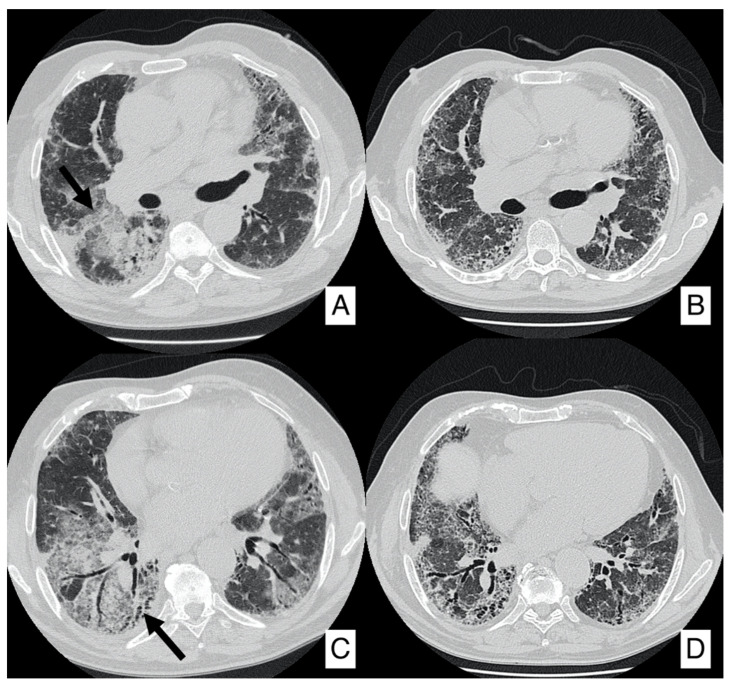
Same IPF patient as Figure 8: comparison between CT acquired during exacerbation (**A**,**C**) and follow-up scan (**B**,**D**), the latter performed 4 months later. Images below the carena (**A**,**B**) demonstrate resolution of ground-glass opacification of the right lung (apical segment of the lower lobe, black arrow); similarly, images acquired at basal levels clearly show that ground-glass attenuation of the lower lobes (black arrow for the right lower lobe) decreased at the follow-up evaluation.

The treatment given to this patient was oxygen therapy and corticosteroid drugs. A new CT, performed four months after the episode of acute exacerbation, demonstrated a reduction in ground-glass opacification in the lung parenchyma (Figure 10). In the last follow-up, in October 2019, wheezes in the bilateral basal-middle areas were depicted at the clinical evaluation and antifibrotic therapy was suggested.

### 4.3. Pulmonary Hypertension

Pulmonary hypertension (PH) is defined as a mean pulmonary arterial pressure (mPAP) of >25 mmHg, in the presence of a normal left atrial pressure, with a pulmonary artery wedge pressure of ≤15 mmHg and elevated pulmonary vascular resistance of >3 wood units with a cardiac index of <2.5 L/min/m^2^ [59]. PH is frequently found in advanced stages of IPF disease or when emphysema is associated, having combined pulmonary fibrosis and emphysema syndrome (CPFE) [60,61,62]. Studies have reported that in the early stages or at diagnosis of IPF disease, 8% to 15% of patients may already have PH, and in advanced IPF, the frequency rises to 32–50% of patients [60,63,64]. Furthermore, comorbidities, such as obstructive sleep apnea, cardiac diastolic dysfunction or pulmonary thromboembolism, may increase the frequency of PH in patients with IPF. According to Simonneau et al., pulmonary hypertension has been classified into 5 groups [65]:

- Group I—which includes idiopathic or hereditable pulmonary arterial hypertension due to the affection of lung vasculature;
- Group II—related to left heart disease;
- Group III—PH associated with chronic lung disease and hypoxemia;
- Group IV—represented by thromboembolic pulmonary hypertension (CTEPH);
- Group V—PH caused by unclear and multifactorial mechanisms.

Considering that PH’s symptoms are similar to those of IPF disease, PH should be suspected in IPF patients with exertional dyspnea, oxygen requirement out of proportion to pulmonary function damage, severe limitation to exercise capacity, decreased DLCO values, evidence of right heart failure on physical exam and evidence of pulmonary artery enlargement or right ventricular hypertrophy on imaging [66,67,68]. It needs to be detected as early as possible in patients with IPF, due to its progressive behavior.

Right heart catheterization (RHC) is the gold standard for the diagnosis, but it is not routinely used as a screening due to its invasiveness [67,69]. Transthoracic echocardiography is usually used to evaluate PH, estimating the right ventricular systolic pressure (RVSP) by the systolic tricuspid regurgitation velocity at doppler. However, in patients with chronic lung, the accuracy of transthoracic echocardiography is reduced, due to a poor acoustic window [70].

CT exams, routinely acquired in IPF patients, may offer the possibility of a pulmonary trunk diameter measurement. Several cut-off values have been suggested: more in detail, a cut off value of >29 mm has been considered quite specific for the diagnostic suspicion of PH. A more recent study suggests a cut off value of >31 mm to obtain greater specificity; however, this threshold has been validated for patients without interstitial diseases [71].

Though, it is necessary for a pulmonary catheterization to have a certain diagnosis.

There is no specific treatment about PH in patients with IPF, but the current ESC/ERS guidelines counsel the use of long-term O_2_ therapy, especially in patients with hypoxemia. Olschewski et al. introduced the use of inhaled Prostacyclin and Iloprost in patients with lung fibrosis by obtaining a reduction in pulmonary pressure [72], while Sildenafil was introduced by Ghofrani et al. [73].

New therapies are going to be introduced thanks to studies based on PH associated with IPF, such as the use of stem/mesenchymal cells and other regenerative therapies used to restore functions of the endothelial cells and reduce the inflammatory and fibrotic processes [63].

**Figure 11:**
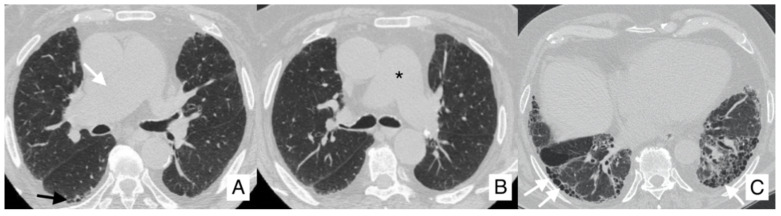
A 77-year-old patient with IPF and pulmonary hypertension (PH). (**A**,**B**) clearly demonstrate the enlargement of the right pulmonary artery (white arrow) and pulmonary trunk (black asterisk). Small cystic areas may be appreciated in the apical segment of the right lower lobe (black arrow). Reticulation bronchiectasis and honeycomb areas are well-depicted in (**C**) (white arrows).

From our case series, we briefly report the clinical history and radiological findings of a 77-year-old patient with IPF and PH. In August 2019, this patient was investigated for disease progression using a CT scan: a comparison with previous studies (acquired from 2016 up to May 2019) was performed. The main imaging findings of the IPF pattern, represented by honeycombing in the basal regions of the lower lobes and in the subpleural areas of the middle level, reticulations, cystic bronchiectasis and para-septal emphysema, were considered stable. During this study, the pulmonary trunk and the right and left pulmonary arteries were depicted as enlarged. The pulmonary trunk diameter is 46 mm, the right pulmonary artery is 42 mm in diameter and the left one is 31 mm in diameter (Figure 11).

### 4.4. Pneumothorax

IPF patients with the development of PNX have a poor prognosis—reporting an overall survival of circa 13 months. There are some risk factors: male sex, tall and thin stature, history of smoking and subpleural bullae [74]. In addition, patients with IPF and PNX may have lower FVC values and rapid deterioration of CT findings [75]. Chest radiography may be negative so that HRCT plays an important role in the recognition of this pathology.

Pneumothorax in IPF patients is often refractory to treatment: this could be explained by the relative rigidity of the lung parenchyma. Suction drainage is ineffective, due to the need of a high negative pressure that is inapplicable in patients with fibrosis [5,76]. Blood-patch and chemical pleurodesis are usually performed, but pneumothorax could occur again. Surgery is preferred as treatment, even if patients may have high post-operative complications and increased risk of mortality, due to exacerbation and infections [74].

From our database, we briefly report the clinical history of a patient with a diagnosis of IPF in 2016, which developed pneumothorax as a main complication. This patient—a 61-year-old man—was studied by CT examinations, acquired firstly at June 2016, and then at the follow-up of September 2018 (Figure 11). The images did not show real changes, confirming the IPF pattern (with diffuse honeycombing, especially in middle-basal lungs, pulmonary reticulations, and bronchiectasis in mild progression) and the presence of bullae of paraseptal emphysema in the bilateral apexes.

**Figure 12:**
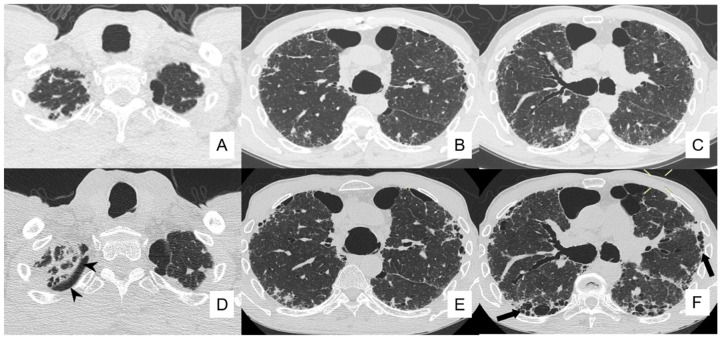
A 61-year-old man with IPF. Images (**A**–**C**) (acquired in 2016) demonstrate CT signs of interstitial disease (reticulations and bronchiectasis). Bullae of paraseptal emphysema in the bilateral apexes are also evident. HRCT images acquired in 2018 (**D**–**F**) identified a minimum flap of pneumothorax (7 mm in thickness, located in right apex).

Compared with the June 2016 CT, the new HRCT images acquired in 2018 identified consolidation areas with irregular margins in the lower part of right upper lobe and a minimum flap of pneumothorax, 7 mm in thickness, located in the right apex (Figure 12).

### 4.5. Pulmonary Infection

Patients with diffuse pulmonary fibrosis are predisposed to the development of pneumonia, and namely to a wide range of pulmonary infections—mainly related to Mycobacterium species, Aspergillus species and Pneumocystis jirovecii pneumonia (PJP) [77,78,79,80]. The underlying fibrosis of the pulmonary parenchyma is associated with high susceptibility for these infections, due to several factors (chronic hypoxemia, etc.); in addition, the increased predisposition is related to the immunosuppressive status of the IPF patients.

Aspergillus infection usually consists of the development of an aspergilloma in areas of preexisting fibro-cavitary disease or may be encountered as chronic necrotizing aspergillosis [79,81]. Aspergilloma may also change its appearance within a lung cavity or dilated bronchus; the inflammatory response—which leads to friable and hypervascular cavity walls—may be responsible for the manifestation of hemoptysis. Chronic necrotizing aspergillosis is depicted as focal consolidation, usually located within the upper lobes: it may, eventually, cavitate [79].

HRCT can be useful to evaluate the presence of complications related to infectious diseases, even if preexisting pulmonary abnormalities (honeycomb, ground-glass opacities, traction bronchiectasis) may mask the radiographical appearances of these infections. HRCT appearances of PJP, in patients with otherwise normal lungs, are those of bilateral, symmetric and extensive ground-glass opacities, consolidations and septal thickening in the lung [82]; the distribution is typically found in perihilar regions and in the upper zones, with sparing of the subpleural lung. However, in IPF patients, it builds to be very difficult to distinguish PJP appearance from acute exacerbation of IPF. Pure ground-glass areas and crazy paving appearances, even if not easily recognizable, represent the most important imaging features.

Furthermore, patients with IPF have a high risk of reactivation of pulmonary tuberculosis (TB) [77,78]. The radiological manifestations of this infection may be the development of peripheral mass-like lesions, that may resemble IPF-associated lung cancer or segmental or lobar consolidation with and without necrotizing cavitation. The very insidious appearance for radiologists is clearly represented by the tuberculoma, which may simulate a neoplastic lesion. It could be very helpful, in these cases, in the identification of a tree-in-bud appearance adjacent to the mass, in order to provide a differential diagnosis. The peribronchial pattern of tree-in-bud nodules—adjacent to the nodule—may increase the diagnostic capability in the differential diagnosis.

**Figure 13:**
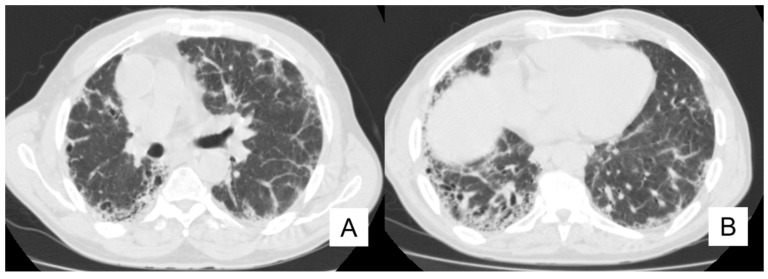
An 80-year-old man with values of SpO_2_, Forced Vital Capacity (FVC) and Forced Expiratory Volume in one second (FEV1) equal to 94%, 57% and 56%, respectively (at first CT scan). A typical IPF pattern was clearly depicted (**A**,**B**).

**Figure 14:**
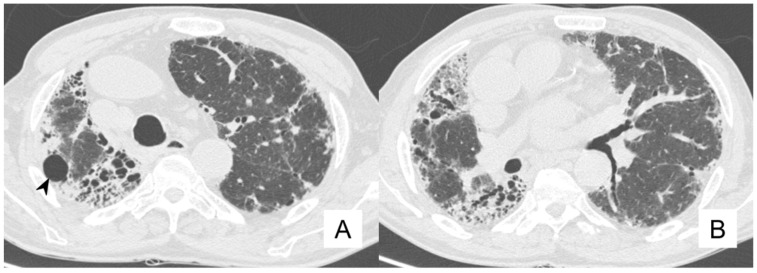
Same patient as the previous figure (Figure 12)—follow-up HRCT performed one year later. Images demonstrate disease progression, with an increased representation of honeycomb and traction bronchiectasis (**A**,**B**). These findings were associated with the development of cystic cavities, namely in the right upper lobe (black arrowhead in **A**).

**Figure 15:**
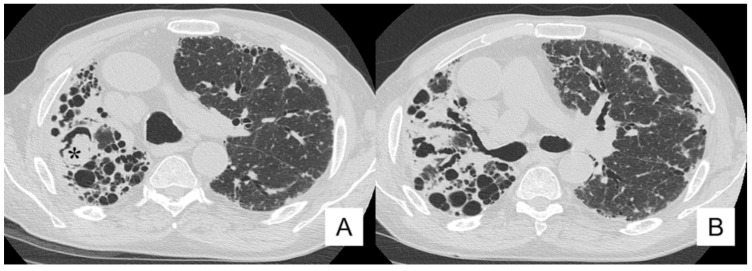
Same patient as previous figures (Figure 12 and Figure 13), HRCT acquired 2 years after the onset of IPF disease, due to clinical worsening (fever and weight loss). In the right upper lobe (**A**,**B**), we have now identified a cystic space containing dense material (black asterisk in **A**), resembling the monod sign. Based on this morphological appearance, the suspicion of mycotic colonization was formulated by the radiologists; after the sputum collection, a final diagnosis of pulmonary aspergilloma was made.

Moreover, we discuss the clinical history and radiological findings of a patient with IPF and mycotic infection. At the onset of the disease, in 2016, a man of 80-years-old, ex-smoker (1 pack-year), reported values of SpO_2_, FVC and FEV1 equal to 94%, 57% and 56%, respectively; at the first HRCT scan, a typical IPF pattern was clearly depicted (Figure 13). The follow-up HRCT, performed one year later, demonstrated disease progression, with an increased representation of honeycomb and traction bronchiectasis (Figure 14). In 2018, he reported a clinical worsening—due to the development of fever and weight loss (about 5 kg); based on the laboratory findings (PCR 9.80), pulmonary function tests (decrease of FVC) and clinical symptoms, the presence of inflammatory and/or infective disease was suspected. The patients were again investigated with a CT scan in our institute, which showed imaging of disease progression, with an increased representation of honeycombing, (especially in the right hemithorax), volume loss and traction bronchiectasis. These findings were associated with the development of cystic cavities, mainly located in the apical regions (Figure 14). In the right upper lobe, we also identified a cystic space containing dense material (Figure 15); based on this morphological appearance, the suspicion of a mycotic colonization was formulated by the radiologists. A final diagnosis of pulmonary aspergilloma was obtained after the sputum collection.

## 5. Conclusions

The knowledge of complications that may be encountered during IPF follow-up is very important for these patients: it concerns not only pneumologists but also radiologists, surgeons and pathologists. In this article, we have focused on clinical and radiological features of complications, in order to help radiologists in their identification on HRCT images. In addition, with the knowledge of the real risk of complications associated with IPF disease, it is very important to address the patient to different diagnostic approaches or therapies, as soon as possible, in order to increase overall survival.
